# Supplementary material for: P4-ATPase control over phosphoinositide membrane asymmetry and neomycin resistance
Source: bioRxiv. 2025 Mar 3:2025.03.03.641220. Preprint. [Version 1] doi: 10.1101/2025.03.03.641220 (PMC11908233; doi:10.1101/2025.03.03.641220)
Supplement: Supplement 1 [file NIHPP2025.03.03.641220v1-supplement-1.pdf]

bioRxiv preprint doi: https://doi.org/10.1101/2025.03.03.641220; this version posted March 3, 2025. The copyright holder for this preprint (which was not certified by peer review) is the author/funder, who has granted bioRxiv a license to display the preprint in perpetuity. It is made available under aCC-BY 4.0 International license.

Supplementary Information

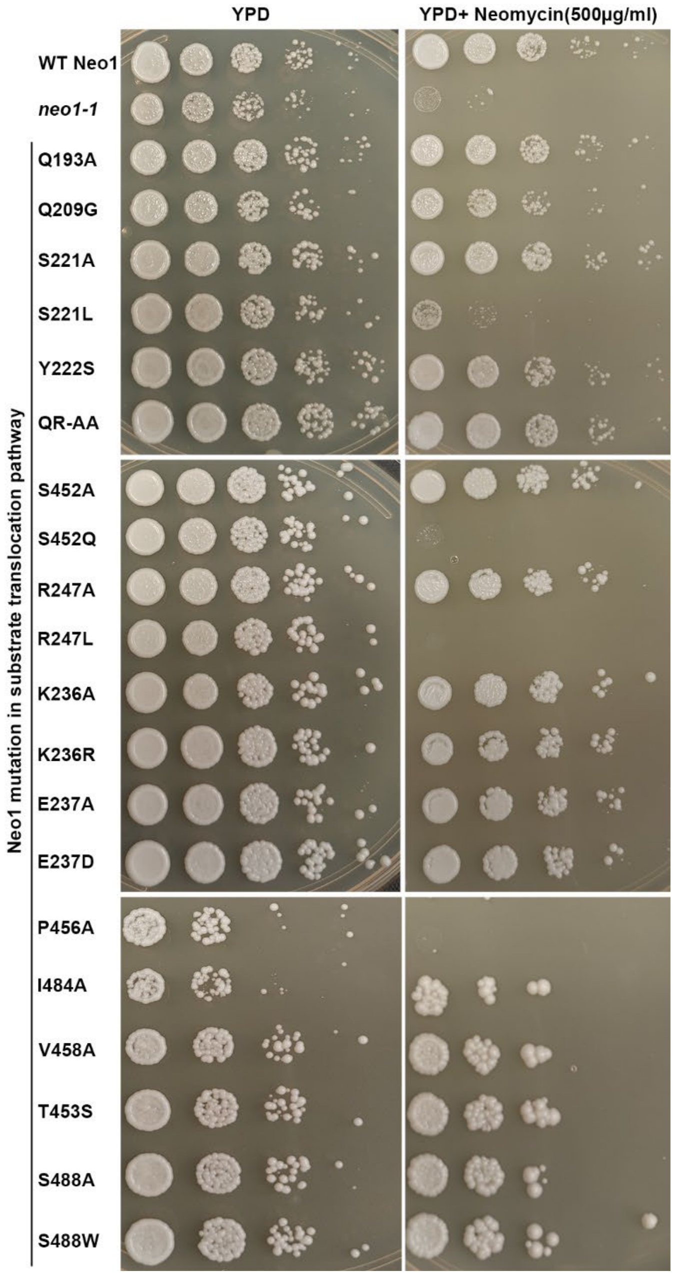

Extended Data Fig. 1: Neo1 substrate transport pathway mutants are sensitive to neomycin. Neomycin sensitivity assay of *neo1* substrate transport pathway mutants at 26 °C on YPD and YPD Neomycin (500 μg/ml) plates.

bioRxiv preprint doi: https://doi.org/10.1101/2025.03.03.641220; this version posted March 3, 2025. The copyright holder for this preprint (which was not certified by peer review) is the author/funder, who has granted bioRxiv a license to display the preprint in perpetuity. It is made available under aCC-BY 4.0 International license.

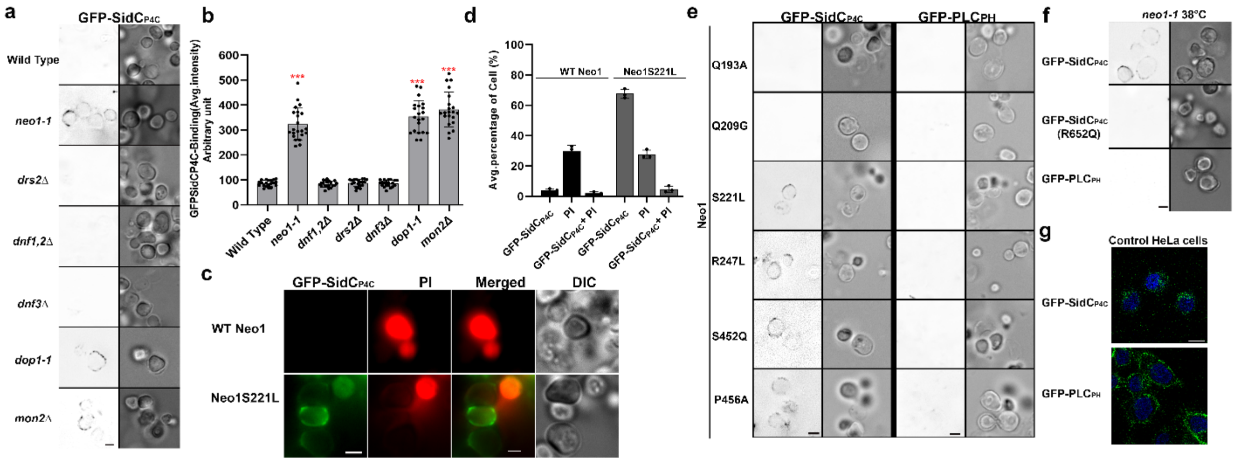

Extended Data Fig. 2: Neomycin sensitive *neo1* substrate transport pathway mutants expose PI4P in extracellular leaflets. a, *neo1-1, dop1-1* and *mon2*Δ mutant exposes PI4P on extracellular leaflets probed using recombinantly purified GFP-SidC$_{P4C}$. The right panel is the fluorescence signal intensity of the GFP-probe and left panel shows the DIC panel to display yeast cells. Scale bar = 2 µm. **b,** Quantification of GFP-SidC$_{P4C}$ binding to the cell surface of the WT cells and flippase mutant strains. One-way ANOVA was performed to test the variance and comparisons with WT cells were made with Tukey's post hoc analysis ($n = 20$, ±standard deviation (SD). P<0.001 is ***. **c,** Intact live cells expose PI4P on the extracellular leaflet. **d,** Quantification of the percentage of the cells exposing PI4P stained with PI. **e,** Perturbation of plasma membrane phosphoinositide asymmetry was tested in *neo1* mutant strains.Scale bar = 2 µm. **f,** Only GFP-SidC$_{P4C}$ binds to neomycin-sensitive mutant cells at the rim of cells, suggesting exposure of PI4P on extracellular leaflet of the plasma membrane. Scale bar = 2 µm. **g,** HeLa cells were fixed, permeabilized and stained with recombinant biosensors GFP-SidC$_{P4C}$ or GFP-PLC$_{PH}$. Scale bar = 10 µm.

bioRxiv preprint doi: https://doi.org/10.1101/2025.03.03.641220; this version posted March 3, 2025. The copyright holder for this preprint (which was not certified by peer review) is the author/funder, who has granted bioRxiv a license to display the preprint in perpetuity. It is made available under aCC-BY 4.0 International license.

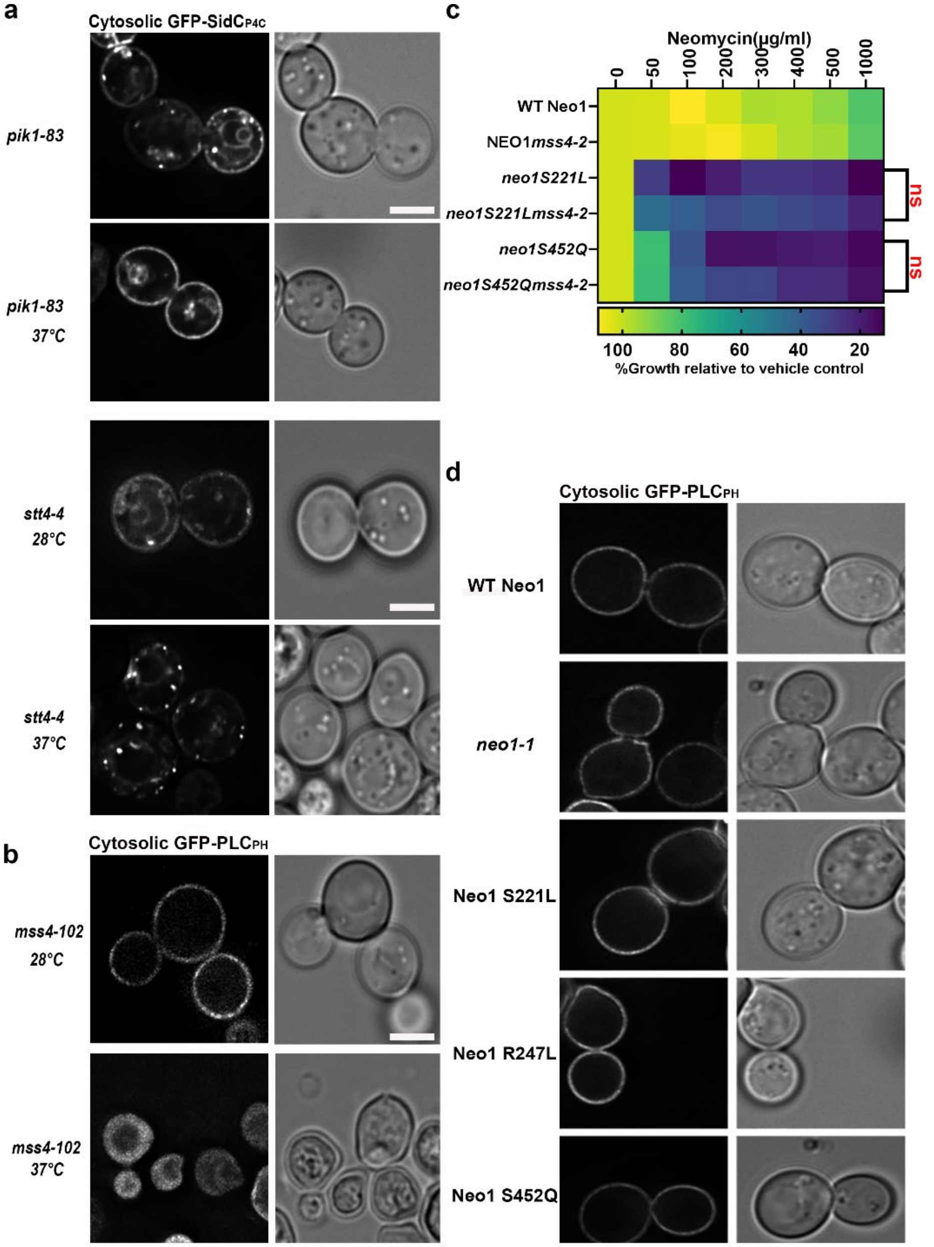

Extended Data Fig. 3: Confirmation of *pik1-83, stt4-4* and *mss4-102* mutants. a, Live cell fluorescence microscopy of intracellular (cytosolic) GFP-SidC$_{P4C}$ in PI kinase mutant cells *pik1-83* and *stt4-4*, at permissive (28 °C) and non-permissive temperature (37 °C). **b,** Neo1 neomycin sensitive mutants do not have any effect on localization of intracellular PI(4,5)P_2 probed with GFP-PLC$_{PH}$ expressed in the yeast cytosol**.** Scale Bar =2 µm. n=3 biological replicates. **c,** A PI 5-kinase Mss4 mutant allele fails to suppress the neomycin sensitivity of *neo1* mutants. The data represent growth relative to WT cells without the drug. Two-way ANOVA was performed to test the variance and comparisons with *neo1S221L* or *neo1S452Q* were made with Tukey's multiple comparisons test (n = 3, ±standard deviation (SD). ns represents non-significant. **d,** localization of GFP-PLC$_{PH}$ expressed in the cytosol of a *mss4-102* mutant at permissive (28 °C) and non-permissive temperature (37 °C) Scale Bar = 2 µm. n=3 biological replicates.

bioRxiv preprint doi: https://doi.org/10.1101/2025.03.03.641220; this version posted March 3, 2025. The copyright holder for this preprint (which was not certified by peer review) is the author/funder, who has granted bioRxiv a license to display the preprint in perpetuity. It is made available under aCC-BY 4.0 International license.

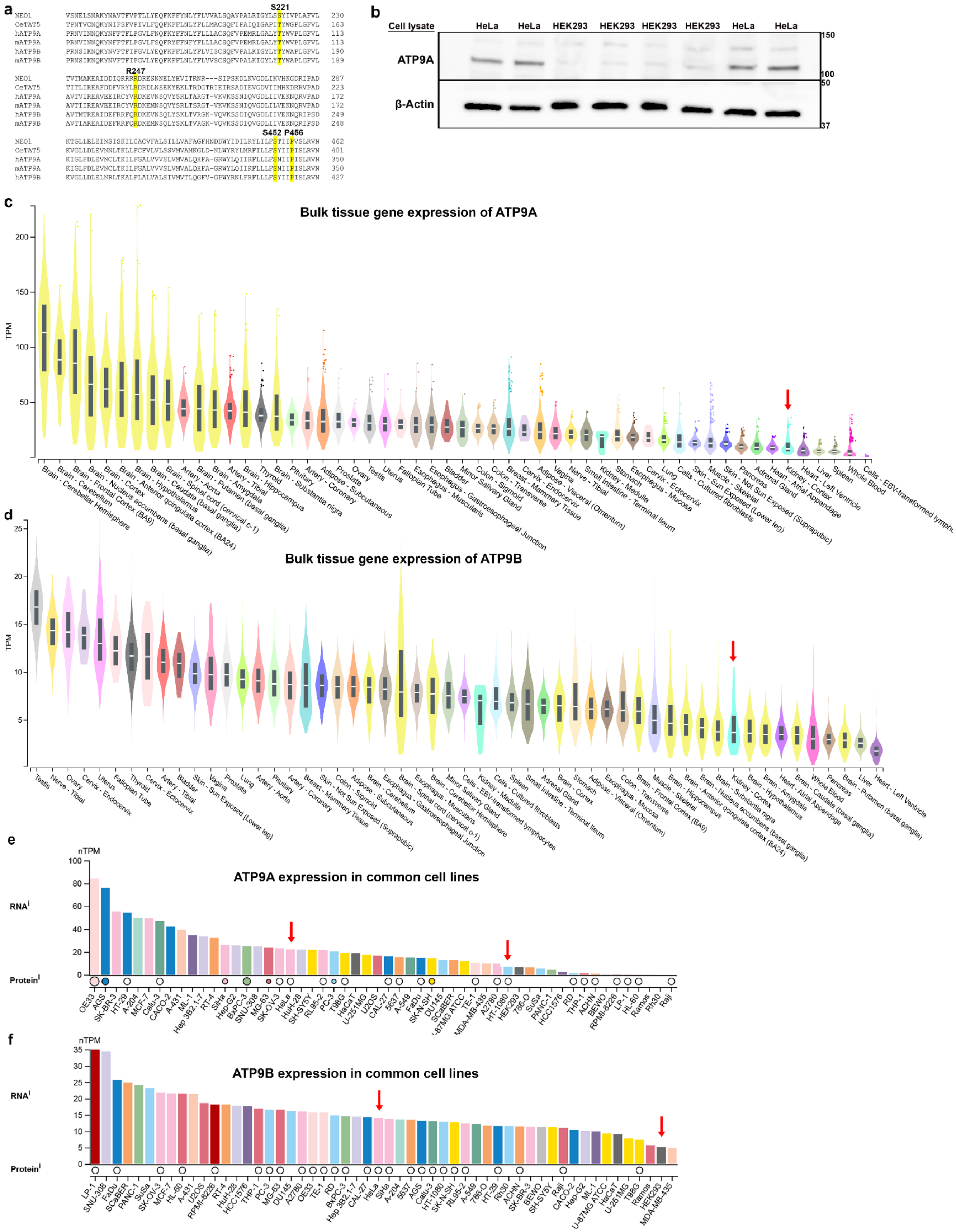

bioRxiv preprint doi: https://doi.org/10.1101/2025.03.03.641220; this version posted March 3, 2025. The copyright holder for this preprint (which was not certified by peer review) is the author/funder, who has granted bioRxiv a license to display the preprint in perpetuity. It is made available under aCC-BY 4.0 International license.

Extended Data Fig.4: Expression of ATP9A and ATP9B: a, Sequence alignment of Neo1 with human and mouse orthologs ATP9A, ATP9B and *C. elegans* Tat5**. b,** Expression of ATP9A in HeLa cells and HEK293 cell lysates by immunoblotting**. c,d** RNA-seq data of ATP9A and ATP9B in tissues. **e,f** RNA- seq data of ATP9A and ATP9B in commonly used cell lines. Arrow highlights the expression of ATP9A in Kidney tissues, HeLa cells and HEK293 cells. The expression plot images were adopted from Genotype-Tissue Expression (GTEx) Portal and Human Protein Atlas (ATP9A: https://www.proteinatlas.org/ENSG00000054793-ATP9A, ATP9B: https://www.proteinatlas.org/ENSG00000166377-ATP9B). The data used for the analyses described in this manuscript were obtained from the GTEx Portal on 01/20/2025.

bioRxiv preprint doi: https://doi.org/10.1101/2025.03.03.641220; this version posted March 3, 2025. The copyright holder for this preprint (which was not certified by peer review) is the author/funder, who has granted bioRxiv a license to display the preprint in perpetuity. It is made available under aCC-BY 4.0 International license.

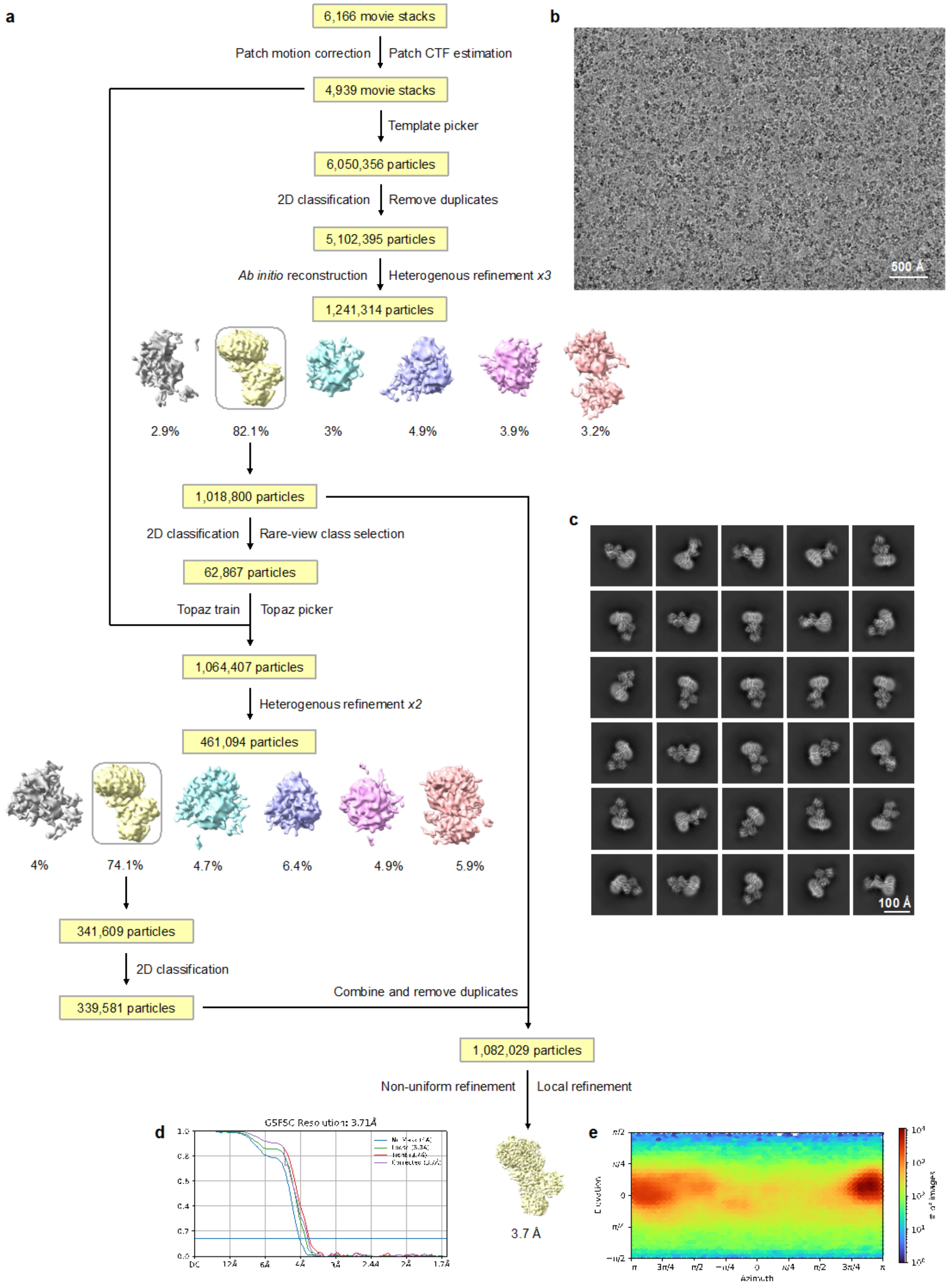

Extended Data Fig. 5: Cryo-EM structural determination of peptidisc-reconstituted Neo1 bound with PI4P in the E2P state. a, Data processing workflow. **b,** Representative raw micrograph. A total of 6,166 such micrographs were recorded. **c,** Representative 2D classes. **d,** Gold-standard Fourier shell correlation (GSFSC) curve for the 3D reconstruction. **e,** Angular distribution heat map for the 3D reconstruction.

bioRxiv preprint doi: https://doi.org/10.1101/2025.03.03.641220; this version posted March 3, 2025. The copyright holder for this preprint (which was not certified by peer review) is the author/funder, who has granted bioRxiv a license to display the preprint in perpetuity. It is made available under aCC-BY 4.0 International license.

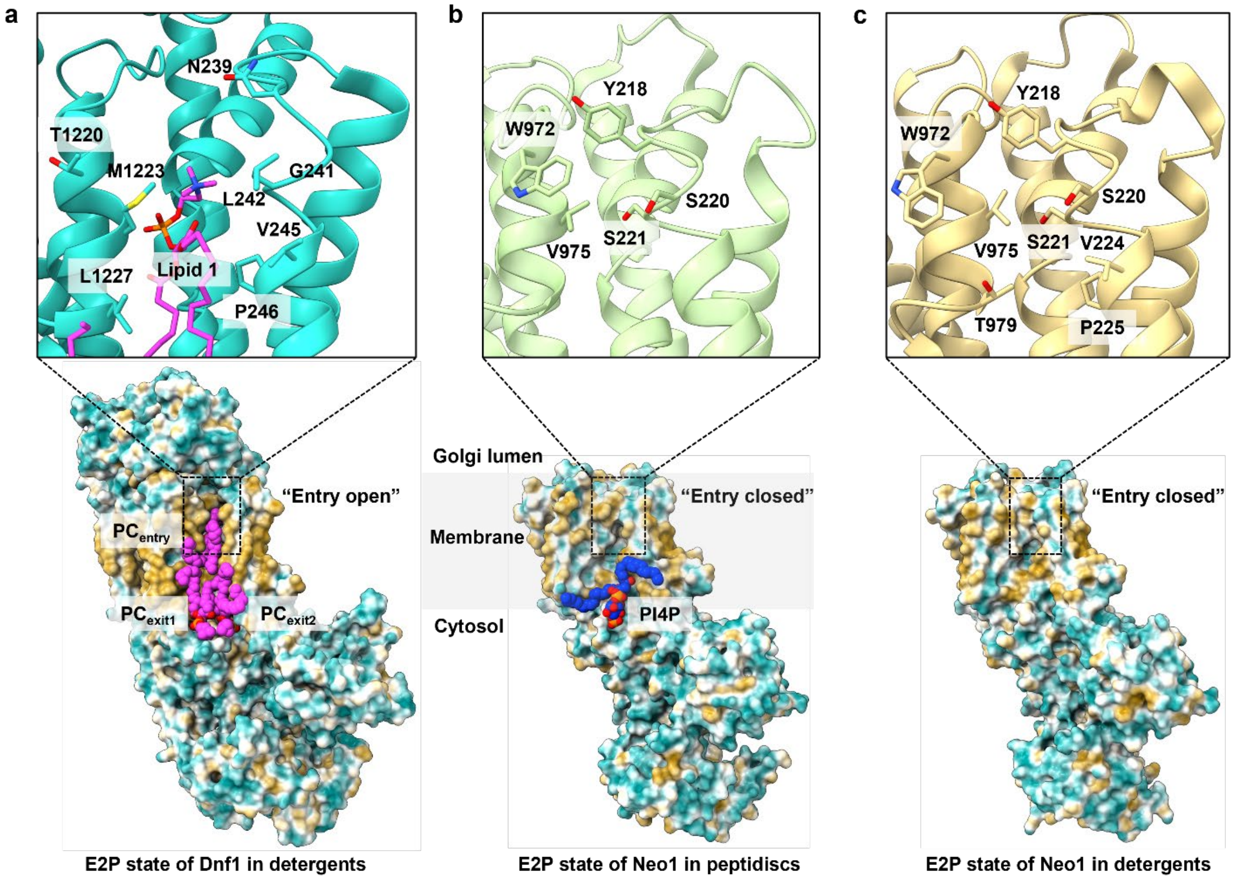

Extended Data Fig. 6: Comparison of substrate binding sites in Neo1 and Dnf1. Substrate entry site is open in the E2P state of Dnf1 in detergents (PDB ID 7KYC) (**a**), but is closed in the peptidisc-reconstituted Neo1 in E2P state (**b**, this study), and in the E2P state of Neo1 in detergents (PDB ID 7RD6) (**c**). Residues surrounding the substrate entry site are shown in sticks. The entry site is more tightly closed in detergent-reconstituted Neo1, involving additional residues T979, V224 and P225.

bioRxiv preprint doi: https://doi.org/10.1101/2025.03.03.641220; this version posted March 3, 2025. The copyright holder for this preprint (which was not certified by peer review) is the author/funder, who has granted bioRxiv a license to display the preprint in perpetuity. It is made available under aCC-BY 4.0 International license.

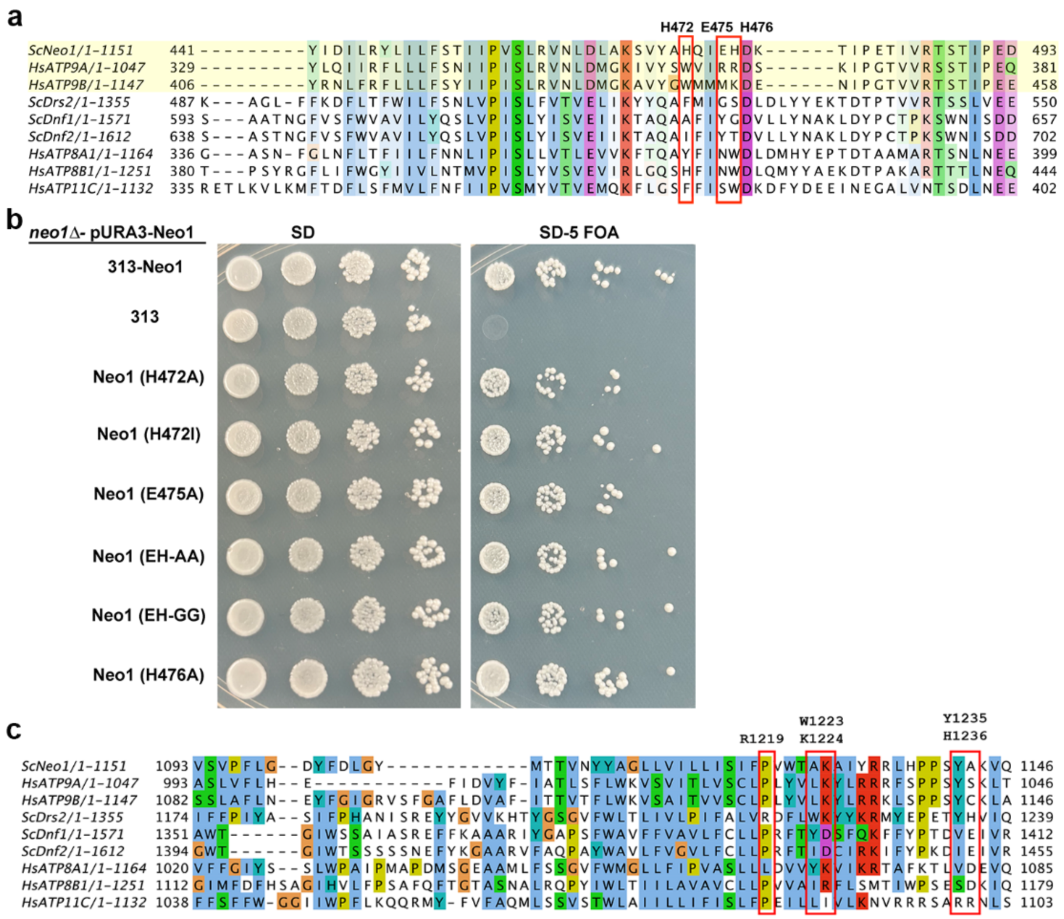

Extended Data Fig.7: Mutation in the Neo1 PI4P binding region does not affect cell growth: **a,** Sequence alignment of Neo1 PI4P binding region with human orthologs ATP9A, ATP9B and Drs2. *Sc*: *Saccharomyces cerevisiae*. *Hs*: *Homo sapiens*. **b,** All of the PI4P binding region Neo1 mutants support viability of *neo1*Δ cells. We transformed a *neo1*ΔpURA3-NEO1 strain with HIS3-marked plasmids harboring the indicated Neo1 variant. Cells were spotted on complete media SD to select both plasmids and on SD-5-FOA plates to pop out pURA3-NEO1 plasmid which will allow the expression on 5-FOA plates. NEO1 is an essential gene, and all Neo1 mutants were able to complement the growth defect of *neo1*Δ strain. **c,** Sequence alignment of Drs2 PI4P binding region with Neo1, ATP9A and ATP9B. The Drs2 PI4P binding site residues are not well conserved in Neo1/ATP9A/ATP9B.

bioRxiv preprint doi: https://doi.org/10.1101/2025.03.03.641220; this version posted March 3, 2025. The copyright holder for this preprint (which was not certified by peer review) is the author/funder, who has granted bioRxiv a license to display the preprint in perpetuity. It is made available under aCC-BY 4.0 International license.

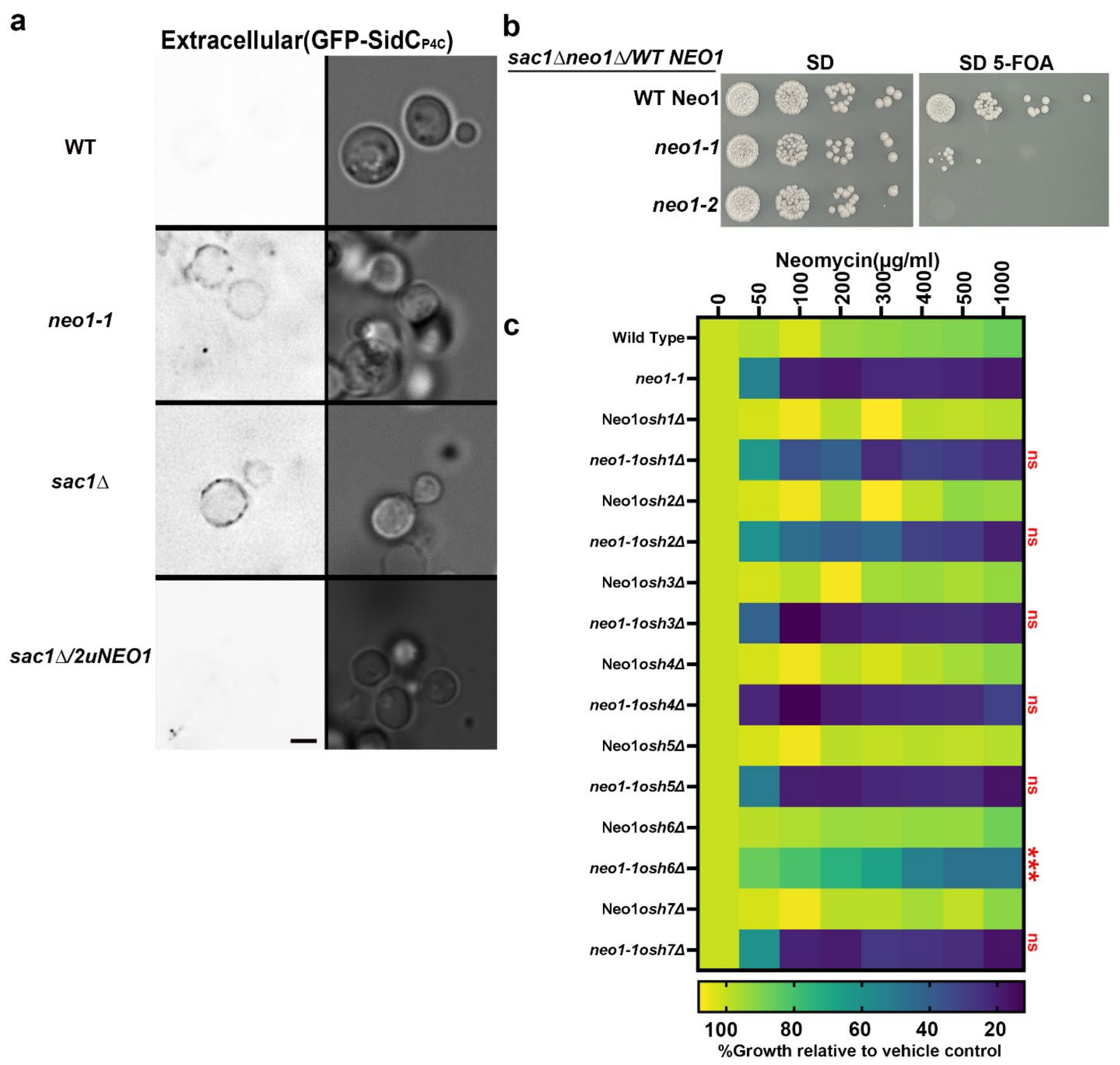

Extended Data Fig. 8: *sac1*Δ cells expose PI4P in extracellular leaflets and neomycin sensitivity assay of *neo1-1osh* mutants. a, *sac1*Δ cells expose PI4P in extracellular leaflets and overexpression of *NEO1* from a 2µ plasmid suppresses the PI4P exposure. The right panel is the fluorescence signal intensity of the GFP-probe and left panel shows the DIC panel to display yeast cells. Scale Bar = 2 µm. n=3 biological replicates. **b,** *neo1* temperature sensitive mutants *(ts) neo1-1 sac1*Δ *or neo1-2sac1*Δ mutants are inviable or grow poorly on 5-FOA plates. We transformed a *sac1*Δ*neo1*ΔpURA3-NEO1 strain with LEU-marked plasmids harboring the indicated Neo1 temperature sensitive variant. Cells were spotted on complete media SD to select both plasmids and on SD-5-FOA plates to pop out pURA3-NEO1 plasmid which will allow the expression of Neo1 variant on 5-FOA plates. **c,** Deletion of only *osh6*Δ suppresses the neomycin sensitivity of *neo1* mutants. The data represent growth relative to WT cells without the drug. Two-way ANOVA was performed to test the variance and comparisons with *neo1-1* were made with Tukey's multiple

bioRxiv preprint doi: https://doi.org/10.1101/2025.03.03.641220; this version posted March 3, 2025. The copyright holder for this preprint (which was not certified by peer review) is the author/funder, who has granted bioRxiv a license to display the preprint in perpetuity. It is made available under aCC-BY 4.0 International license.

comparison test (n = 3, ±standard deviation (SD). ns represents non-significant. *** represents P<0.001.

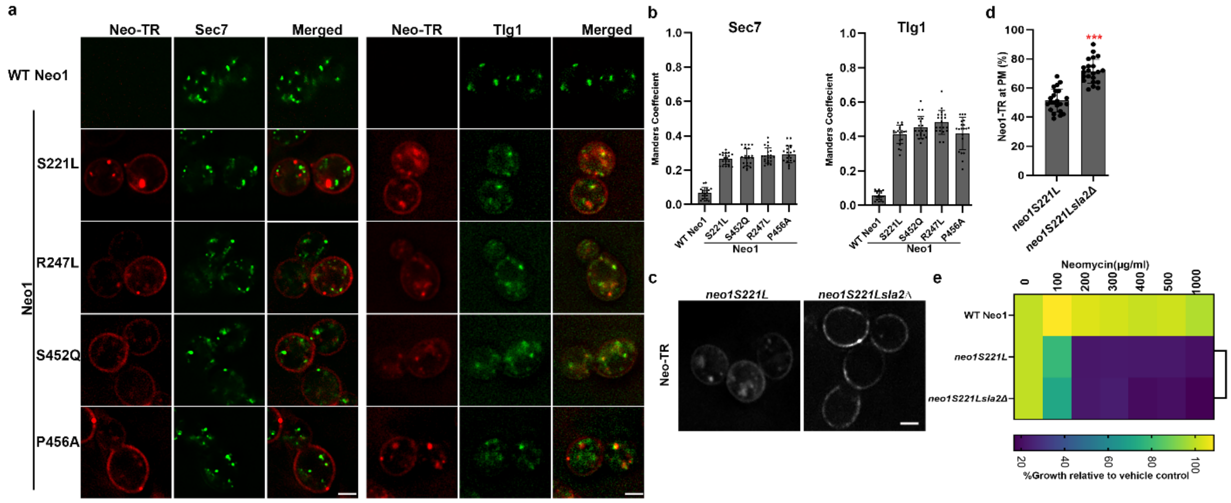

Extended Data Fig. 9: Neomycin binds to exposed PI4P and is endocytosed. a, Neo-TR binds neomycin-sensitive *neo1* mutants and endocytosed to the *trans*-Golgi Network (Sec7) and endosomal compartment (Tlg1). **b,** Colocalization of Neo-TR with TGN marker Sec7 and endosomal marker Tlg1 n=20 cells. **c,** knockout of *sla2*Δ in neomycin sensitive mutant *neo1 S221L* blocks Neo-TR internalization. **d,** Quantification of Neo-TR fluorescence intensity at the PM. For all quantification, data from ~20 cells from three independent experiments were obtained and analyzed. Scale bar = 2 µm **e,** Deletion of *sla2*Δ fails to suppress the neomycin sensitivity of neo1 mutants Neo1 S221L. The data represent growth relative to WT cells without the drug. Two-way ANOVA was performed to test the variance and comparisons with *neo1S221L* were made with Tukey's post hoc analysis (n = 3, ±standard deviation (SD). ns represents non-significant.

bioRxiv preprint doi: https://doi.org/10.1101/2025.03.03.641220; this version posted March 3, 2025. The copyright holder for this preprint (which was not certified by peer review) is the author/funder, who has granted bioRxiv a license to display the preprint in perpetuity. It is made available under aCC-BY 4.0 International license.

Extended Data Table 1. Cryo-EM data collection, refinement and validation statistics

	Neo1 in E2P state bound with PI4P (EMD-44850) (PDB 9BS1)
**Data collection and processing**	
Magnification	105,000
Voltage (keV)	300
Electron dose (e^-/Å^2)	58
Defocus range (-µm)	1.3-1.6
Pixel size (Å)	0.828
Symmetry imposed	C1
Initial particle images (no.)	6,050,356
Final particle images (no.)	1,082,029
Map resolution (Å)	3.7
FSC threshold	0.143
Map resolution range (Å)	1.8-10.6
**Refinement**	
Initial model used (PDB code)	AF-P40527-F1-model_v4
Model resolution (Å)	4.0
FSC threshold	0.5
Map sharpening *B* factor (Å^2)	-175.3
Model composition	
Non-hydrogen atoms	7,757
Protein residues	965
Ligands	2
B factors (Å^2)	
Protein	69.42
Ligand	20.00
R.m.s. deviations	
Bond lengths (Å)	0.003
Bond angles (°)	0.621
**Validation**	
MolProbity score	1.98
Clashscore	9.52
Poor rotamers (%)	0.23
Ramachandran plot	
Favored (%)	92.20
Allowed (%)	7.80
Disallowed (%)	0.00

bioRxiv preprint doi: https://doi.org/10.1101/2025.03.03.641220; this version posted March 3, 2025. The copyright holder for this preprint (which was not certified by peer review) is the author/funder, who has granted bioRxiv a license to display the preprint in perpetuity. It is made available under aCC-BY 4.0 International license.

Extended Data Table 2: Yeast strains and plasmids used in the study.

Strain	Genotype	Plasmid	Source
BKJ424	MATa *his3Δ1 leu2Δ0* ura3Δ0 lys2Δ0*neo1Δ*::KanMX	pRS313-NEO1	[1]
BKJ426	MATa *his3Δ1 leu2Δ0* ura3Δ0 lys2Δ0*neo1Δ*::KanMX	pRS313-NEO1 Q209G	[1]
BKJ427	MATa *his3Δ1 leu2Δ0* ura3Δ0 lys2Δ0*neo1Δ*::KanMX	pRS313-Neo1 S221A	[1]
BKJ428	MATa *his3Δ1 leu2Δ0* ura3Δ0 lys2Δ0*neo1Δ*::KanMX	pRS313-Neo1 S221L	[1]
BKJ429	MATa *his3Δ1 leu2Δ0* ura3Δ0 lys2Δ0*neo1Δ*::KanMX	pRS313-Neo1 S452A	[1]
BKJ430	MATa *his3Δ1 leu2Δ0* ura3Δ0 lys2Δ0*neo1Δ*::KanMX	pRS313-Neo1 S452Q	[1]
BKJ432	MATa *his3Δ1 leu2Δ0* ura3Δ0 lys2Δ0*neo1Δ*::KanMX	pRS313-Neo1 T453S	[1]
BKJ433	MATa *his3Δ1 leu2Δ0* ura3Δ0 lys2Δ0*neo1Δ*::KanMX	pRS313-Neo1 K236A	[1]
BKJ434	MATa *his3Δ1 leu2Δ0* ura3Δ0 lys2Δ0*neo1Δ*::KanMX	pRS313-Neo1 K236R	[1]
BKJ435	MATa *his3Δ1 leu2Δ0* ura3Δ0 lys2Δ0*neo1Δ*::KanMX	pRS313-Neo1 E237A	[1]
BKJ436	MATa *his3Δ1 leu2Δ0* ura3Δ0 lys2Δ0*neo1Δ*::KanMX	pRS313-Neo1 E237D	[1]
BKJ437	MATa *his3Δ1 leu2Δ0* ura3Δ0 lys2Δ0*neo1Δ*::KanMX	pRS313-Neo1 R247A	[1]
BKJ438	MATa *his3Δ1 leu2Δ0* ura3Δ0 lys2Δ0*neo1Δ*::KanMX	pRS313-Neo1 R247L	[1]
BKJ439	MATa *his3Δ1 leu2Δ0* ura3Δ0 lys2Δ0*neo1Δ*::KanMX	pRS313-Neo1 S488A	[1]
BKJ440	MATa *his3Δ1 leu2Δ0* ura3Δ0 lys2Δ0*neo1Δ*::KanMX	pRS313-Neo1 S488W	[1]
BKJ441	MATa *his3Δ1 leu2Δ0* ura3Δ0 lys2Δ0*neo1Δ*::KanMX	pRS313-Neo1 Q193A	[1]
BKJ443	MATa *his3Δ1 leu2Δ0* ura3Δ0 lys2Δ0*neo1Δ*::KanMX	pRS313-Neo1 P456A	[1]
BKJ481	MATa *his3Δ1 leu2Δ0* ura3Δ0 lys2Δ0*neo1Δ*::KanMX	pRS313-Neo1 I484A	This study
BKJ482	MATa *his3Δ1 leu2Δ0* ura3Δ0 lys2Δ0*neo1Δ*::KanMX	pRS313-Neo1 V485A	This study
BY4741	*MATa his3 leu2 ura3 met15*		Invitrogen
ZHY615M2D	*MATα his3 leu2 ura3 lys2 drs2Δ*		[2]
PFY3275F	MATa his3 leu2 ura3 met15 *dnf1Δ dnf2Δ*		[2]

bioRxiv preprint doi: https://doi.org/10.1101/2025.03.03.641220; this version posted March 3, 2025. The copyright holder for this preprint (which was not certified by peer review) is the author/funder, who has granted bioRxiv a license to display the preprint in perpetuity. It is made available under aCC-BY 4.0 International license.

BY4741 YMR162C	*MATa his3 leu2 ura3 met15 dnf3Δ*		Invitrogen
ZHY628-15B	MATα his3 leu2 ura3 *neo1Δ* p413-neo*1-1*		[3,4]
TSA854	MATa dop1-1::kanMX his3Δ1 leu2Δ0 ura3Δ0 met15Δ0 CAN1+ LYP1+		[5]
Y14541	MAT@ mon2Δ::kanMX his3Δ1 leu2Δ0 ura3Δ0 lys2Δ0 + [GALpr-MON2, URA3]		[5]
AAY102	SEY6210; *stt4Δ*::*HIS3* carrying pRS415*stt4-4* (*LEU2 CEN6 stt4-4*)		[6]
BKJ810	SEY6210; *stt4Δ*::*HIS3 neo1Δ* pRS414-Neo1S221L carrying pRS415*stt4-4* (*LEU2 CEN6 stt4-4*)	pRS314-Neo1 S221L	This study
BKJ811	SEY6210; *stt4Δ*::*HIS3 neo1Δ* pRS414-Neo1S452QLcarrying pRS415*stt4-4* (*LEU2 CEN6 stt4-4*)	pRS314-Neo1 S221L	This study
BKJ812	SEY6210; *stt4Δ*::*HIS3 neo1Δ* pRS414-Neo1carrying pRS415*stt4-4* (*LEU2 CEN6 stt4-4*)	pRS314-Neo1	This study
AAY104	SEY6210; pik1Δ::HIS3 carrying pRS314pik1-83 (TRP1 CEN6 pik1-83)		[6]
BKJ813	SEY6210; pik1Δ::HIS3 carrying pRS314pik1-83 (TRP1 CEN6 pik1-83)	pRS315-Neo1	This study
BKJ814	SEY6210; pik1Δ::HIS3 carrying pRS314pik1-83 (TRP1 CEN6 pik1-83)	pRS315-Neo1S221L	This study
BKJ815	SEY6210; pik1Δ::HIS3 carrying pRS314pik1-83 (TRP1 CEN6 pik1-83)	pRS315-Neo1S452Q	This study
AAY202	SEY6210;*mss4Δ::HIS3MX6* YC*plac*111*mss4*^{ts}*-102* (*LEU2 CEN6 mss4*^{ts}*-102*)		[7]
BKJ816	SEY6210;*mss4Δ::HIS3MX6* YC*plac*111*mss4*^{ts}*-102* (*LEU2 CEN6 mss4*^{ts}*-102*)	pRS314-Neo1	This study
BKJ817	SEY6210;*mss4Δ::HIS3MX6* YC*plac*111*mss4*^{ts}*-102* (*LEU2 CEN6 mss4*^{ts}*-102*)	pRS314-Neo1S221L	This study
BKJ818	SEY6210;*mss4Δ::HIS3MX6* YC*plac*111*mss4*^{ts}*-102* (*LEU2 CEN6 mss4*^{ts}*-102*)	pRS314-Neo1S452Q	This study
BKJ819	SEY6210; *stt4Δ*::*HIS3* carrying pRS415*stt4-4* (*LEU2 CEN6 stt4-4*)	pRS416-GFP-SidC$_{P4C}$	This study
BKJ820	SEY6210; pik1Δ::HIS3 carrying pRS314pik1-83 (TRP1 CEN6 pik1-83)	pRS416-GFP-SidC$_{P4C}$	This study

bioRxiv preprint doi: https://doi.org/10.1101/2025.03.03.641220; this version posted March 3, 2025. The copyright holder for this preprint (which was not certified by peer review) is the author/funder, who has granted bioRxiv a license to display the preprint in perpetuity. It is made available under aCC-BY 4.0 International license.

BKJ821	SEY6210; *mss4Δ::HIS3MX6* YC*plac*111*mss4^{ts}-102* (*LEU2 CEN6 mss4^{ts}-102*)	pRS416-GFP-SidC$_{P4C}$	This study
BKJ822	*MATa his3 leu2 ura3 met15 sac1Δ*::NAT		This study
BKJ823	*MATa his3 leu2 ura3 met15 sac1Δ*::NAT	pRS423	
BKJ824	*MATa his3 leu2 ura3 met15 sac1Δ*::NAT	pRS423-Neo1	This study
YWY10	MATa *his3Δ1 leu2Δ0* ura3Δ0 lys2Δ0*neo1Δ*::KanMX	pRS416-Neo1	This study
BKJ825	MATa *his3Δ1 leu2Δ0* ura3Δ0 lys2Δ0*neo1Δ*::KanMX *sac1Δ*:HIS3MX6	pRS416-Neo1	This study
BKJ826	MATa *his3Δ1 leu2Δ0* ura3Δ0 lys2Δ0*neo1Δ*::KanMX *sac1Δ*:HIS3MX6	pRS416-Neo1 pRS315-Neo1	This study
BKJ827	MATa *his3Δ1 leu2Δ0* ura3Δ0 lys2Δ0*neo1Δ*::KanMX *sac1Δ*:HIS3MX6	pRS416-Neo1 pRS315-*neo1-1*	This study
BKJ828	MATa *his3Δ1 leu2Δ0* ura3Δ0 lys2Δ0*neo1Δ*::KanMX *sac1Δ*:HIS3MX6	pRS416-Neo1 pRS315-*neo1-2*	This study
BY4741*osh1Δ*	*MATa his3 leu2 ura3 met15 osh1Δ*		Invitrogen
BY4741*osh2Δ*	*MATa his3 leu2 ura3 met15 osh2Δ*		Invitrogen
BY4741*osh3Δ*	*MATa his3 leu2 ura3 met15 osh3Δ*		Invitrogen
BY4741*osh5Δ*	*MATa his3 leu2 ura3 met15 osh5Δ*		Invitrogen
BY4741*osh6Δ*	*MATa his3 leu2 ura3 met15 osh6Δ*		Invitrogen
BY4741*osh7Δ*	*MATa his3 leu2 ura3 met15 osh7Δ*		Invitrogen
BY4741*osh4Δ*	*MATa his3 leu2 ura3 met15 osh4Δ*		Invitrogen
BMY046a	ZHY628-15B (*neo1-1*) *osh4*Δ::LEU2		
BMY043a	ZHY907-5E (*neo1*Δ pNEO1::URA3) *osh4*Δ::LEU2		
BKJ829	MATa *his3Δ1 leu2Δ0* ura3Δ0 lys2Δ0*neo1Δ*::KanMX *osh6Δ*:HIS3MX6	pRS315-Neo1	This study
BKJ830	MATa *his3Δ1 leu2Δ0* ura3Δ0 lys2Δ0*neo1Δ*::KanMX *osh6Δ*:HIS3MX6	pRS315-Neo1S221L	This study
BKJ831	MATa *his3Δ1 leu2Δ0* ura3Δ0 lys2Δ0*neo1Δ*::KanMX *osh6Δ*:HIS3MX6	pRS315-Neo1S452Q	This study
BKJ832	MATa *his3Δ1 leu2Δ0* ura3Δ0 lys2Δ0*neo1Δ*::KanMX *osh6Δ*:HIS3MX6	pRS315-*neo1-1*	This study
BKJ833	MATa *his3Δ1 leu2Δ0* ura3Δ0 lys2Δ0*neo1Δ*::KanMX *osh1Δ*:HIS3MX6	pRS315-*neo1-1*	This study
BKJ834	MATa *his3Δ1 leu2Δ0* ura3Δ0 lys2Δ0*neo1Δ*::KanMX *osh2Δ*:HIS3MX6	pRS315-*neo1-1*	This study
BKJ835	MATa *his3Δ1 leu2Δ0* ura3Δ0 lys2Δ0*neo1Δ*::KanMX *osh3Δ*:HIS3MX6	pRS315-*neo1-1*	This study
BKJ836	MATa *his3Δ1 leu2Δ0* ura3Δ0 lys2Δ0*neo1Δ*::KanMX *osh5Δ*:HIS3MX6	pRS315-*neo1-1*	This study

bioRxiv preprint doi: https://doi.org/10.1101/2025.03.03.641220; this version posted March 3, 2025. The copyright holder for this preprint (which was not certified by peer review) is the author/funder, who has granted bioRxiv a license to display the preprint in perpetuity. It is made available under aCC-BY 4.0 International license.

BKJ837	MATa *his3Δ1 leu2Δ0* ura3Δ0 lys2Δ0*neo1Δ*::KanMX *osh7Δ*:HIS3MX6	pRS315-*neo1-1*	This study
sec12-4 neo1-1	*MATα leu2 ura3 his3 trp1 lys2 sec12-4 neo1-1::HIS3-KanMX*		[3]
sec14-1 neo1-1	*MATa leu2 his3 lys2 sec14-1 neo1-1::HIS3-KanMX*		[3]
sec18-1 neo1-1	*MATα ura3 leu2 his3 trp1 lys2 suc2 sec18-1 neo1-1::HIS3-KanMX*		[3]
sec21-1 neo1-1	*MATa leu2 ura3 his3 trp1 suc2 sec21-1 neo1-1::HIS3-KanMX*		[3]
BKJ838	MATa his3Δ1 leu2Δ0 ura3Δ0 lys2Δ0neo1Δ::KanMX	pRS313-Neo1 H472A	This study
BKJ839	MATa his3Δ1 leu2Δ0 ura3Δ0 lys2Δ0neo1Δ::KanMX	pRS313-Neo1 H472I	This study
BKJ840	MATa his3Δ1 leu2Δ0 ura3Δ0 lys2Δ0neo1Δ::KanMX	pRS313-Neo1 E475A	This study
BKJ841	MATa his3Δ1 leu2Δ0 ura3Δ0 lys2Δ0neo1Δ::KanMX	pRS313-Neo1 H476A	This study
BKJ842	MATa his3Δ1 leu2Δ0 ura3Δ0 lys2Δ0neo1Δ::KanMX	pRS313-Neo1 EH-AA	This study
BKJ843	MATa his3Δ1 leu2Δ0 ura3Δ0 lys2Δ0neo1Δ::KanMX	pRS313-Neo1 EH-GG	This study
BKJ844	*MATa his3Δ1 leu2Δ0 ura3Δ0 lys2Δ 0neo1Δ::KanMX slaΔ::HIS3*	pRS315-Neo1S221L	This study
BKJ845	MATa his3Δ1 leu2Δ0 ura3Δ0 lys2Δ0neo1Δ::KanMX Sec7-3xGFP	pRS313-Neo1	This study
BKJ846	MATa his3Δ1 leu2Δ0 ura3Δ0 lys2Δ0neo1Δ::KanMX Sec7-3xGFP	pRS313-Neo1 S221L	This study
BKJ847	MATa his3Δ1 leu2Δ0 ura3Δ0 lys2Δ0neo1Δ::KanMX Sec7-3xGFP	pRS313-Neo1 R247L	This study
BKJ848	MATa his3Δ1 leu2Δ0 ura3Δ0 lys2Δ0neo1Δ::KanMX Sec7-3xGFP	pRS313-Neo1 S452Q	This study
BKJ849	MATa his3Δ1 leu2Δ0 ura3Δ0 lys2Δ0neo1Δ::KanMX Sec7-3xGFP	pRS313-Neo1 P456A	This study
BKJ850	MATa his3Δ1 leu2Δ0 ura3Δ0 lys2Δ0neo1Δ::KanMX iGFP-Tlg1	pRS313-Neo1	This study
BKJ851	MATa his3Δ1 leu2Δ0 ura3Δ0 lys2Δ0neo1Δ::KanMX iGFP-Tlg1	pRS313-S221L	This study
BKJ852	MATa his3Δ1 leu2Δ0 ura3Δ0 lys2Δ0neo1Δ::KanMX iGFP-Tlg1	pRS313-Neo1 R247L	This study
BKJ853	MATa his3Δ1 leu2Δ0 ura3Δ0 lys2Δ0neo1Δ::KanMX iGFP-Tlg1	pRS313-Neo1 S452Q	This study
BKJ854	MATa his3Δ1 leu2Δ0 ura3Δ0 lys2Δ0neo1Δ::KanMX iGFP-Tlg1	pRS313-Neo1 P456A	This study

bioRxiv preprint doi: https://doi.org/10.1101/2025.03.03.641220; this version posted March 3, 2025. The copyright holder for this preprint (which was not certified by peer review) is the author/funder, who has granted bioRxiv a license to display the preprint in perpetuity. It is made available under aCC-BY 4.0 International license.

Plasmids used in the study for Bacterial expression:

Plasmid	Source
pET28a-GFPSidC(614-743)	This Study
pET28a-GFPSidC(614-743)R652Q	This Study
His6-EGFP-PH-PLCD1 (Addgene Plasmid #183675)	[8]
YIplac211-iGFP-TLG1 (Addgene Plasmid # 105261)	[9]

References:

1. Bai, L. *et al.* Structural basis of the P4B ATPase lipid flippase activity. *Nat. Commun.* **12**, 5963 (2021).

2. Hua, Z., Fatheddin, P. & Graham, T. R. An essential subfamily of Drs2p-related P-type ATPases is required for protein trafficking between Golgi complex and endosomal/vacuolar system. *Mol. Biol. Cell* **13**, 3162–3177 (2002).

3. Hua, Z. & Graham, T. R. Requirement for neo1p in retrograde transport from the Golgi complex to the endoplasmic reticulum. *Mol. Biol. Cell* **14**, 4971–4983 (2003).

4. Takar, M., Wu, Y. & Graham, T. R. The Essential Neo1 Protein from Budding Yeast Plays a Role in Establishing Aminophospholipid Asymmetry of the Plasma Membrane. *J. Biol. Chem.* **291**, 15727–15739 (2016).

5. van Leeuwen, J. *et al.* Exploring genetic suppression interactions on a global scale. *Science* **354**, (2016).

6. Audhya, A., Foti, M. & SD, E. Distinct roles for the yeast phosphatidylinositol 4-kinases,

bioRxiv preprint doi: https://doi.org/10.1101/2025.03.03.641220; this version posted March 3, 2025. The copyright holder for this preprint (which was not certified by peer review) is the author/funder, who has granted bioRxiv a license to display the preprint in perpetuity. It is made available under aCC-BY 4.0 International license.

Stt4p and Pik1p, in secretion, cell growth, and organelle membrane dynamics. *Mol. Biol. Cell* **11**, 2673–2689 (2000).

7. Audhya, A. & SD, E. Stt4 PI 4-kinase localizes to the plasma membrane and functions in the Pkc1-mediated MAP kinase cascade. *Dev. Cell* **2**, 593–605 (2002).

8. Walpole, G. F. W. *et al.* Kinase-independent synthesis of 3-phosphorylated phosphoinositides by a phosphotransferase. *Nat. Cell Biol.* **24**, 708–722 (2022).

9. Day, K. J., Casler, J. C. & Glick, B. S. Budding Yeast Has a Minimal Endomembrane System. *Dev. Cell* **44**, 56-72.e4 (2018).
